# Evaluation of p21 promoter for interleukin 12 radiation induced transcriptional targeting in a mouse tumor model

**DOI:** 10.1186/1476-4598-12-136

**Published:** 2013-11-12

**Authors:** Urska Kamensek, Gregor Sersa, Maja Cemazar

**Affiliations:** 1 Institute of Oncology Ljubljana, Ljubljana, Slovenia; 2 University of Primorska, Faculty of Health Sciences, Izola, Slovenia

**Keywords:** Gene therapy, Transcriptional targeting, p21 promoter, Interleukin 12, Mouse tumor model, Radiotherapy, Plasmid DNA, Gene electrotransfer

## Abstract

**Background:**

Radiation induced transcriptional targeting is a gene therapy approach that takes advantage of the targeting abilities of radiotherapy by using radio inducible promoters to spatially and temporally limit the transgene expression. Cyclin dependent kinase inhibitor 1 (CDKN1A), also known as p21, is a crucial regulator of the cell cycle, mediating G1 phase arrest in response to a variety of stress stimuli, including DNA damaging agents like irradiation. The aim of the study was to evaluate the suitability of the p21 promoter for radiation induced transcriptional targeting with the objective to test the therapeutic effectiveness of the combined radio-gene therapy with p21 promoter driven therapeutic gene interleukin 12.

**Methods:**

To test the inducibility of the p21 promoter, three reporter gene experimental models with green fluorescent protein (GFP) under the control of p21 promoter were established by gene electrotransfer of plasmid DNA: stably transfected cells, stably transfected tumors, and transiently transfected muscles. Induction of reporter gene expression after irradiation was determined using a fluorescence microplate reader *in vitro* and by non-invasive fluorescence imaging using fluorescence stereomicroscope *in vivo*. The antitumor effect of the plasmid encoding the p21 promoter driven interleukin 12 after radio-gene therapy was determined by tumor growth delay assay and by quantification of intratumoral and serum levels of interleukin 12 protein and intratumoral concentrations of interleukin 12 mRNA.

**Results:**

Using the reporter gene experimental models, p21 promoter was proven to be inducible with radiation, the induction was not dose dependent, and it could be re-induced. Furthermore radio-gene therapy with interleukin 12 under control of the p21 promoter had a good antitumor therapeutic effect with the statistically relevant tumor growth delay, which was comparable to that of the same therapy using a constitutive promoter.

**Conclusions:**

In this study p21 promoter was proven to be a suitable candidate for radiation induced transcriptional targeting. As a proof of principle the therapeutic value was demonstrated with the radio-inducible interleukin 12 plasmid providing a synergistic antitumor effect to radiotherapy alone, which makes this approach feasible for the combined treatment with radiotherapy.

## Background

Radiation induced transcriptional targeting is an approach that uses radiation inducible promoters to achieve spatial as well as temporal control over transgene expression. The approach was made possible by the latest improvements in the physical targeting of radiotherapy [1] and understanding of the molecular mechanisms involved in the cellular response to ionizing radiation [2,3]. One of the promoters used for the approach is that of the *CDKN1A* gene [4,5], encoding the cyclin-dependent kinase inhibitor 1A protein, more commonly known as p21 or also as WAF1 or Cip1. p21 is a crucial regulator of the cell cycle, mediating cell cycle G1 phase arrest in response to stress, and plays a role in cell death, DNA repair, senescence, aging and induced pluripotent stem cells reprograming [6]. Promoter of *p21* gene can be activated through p53-dependent [7] and also p53-independent way by various extrinsic stress stimuli including DNA damaging agents like irradiation and chemotherapeutic drugs, hypoxia and other intrinsic and oncogene stresses [8,9].

The utilization of p21 promoter for radiation induced transcriptional targeting was so far confirmed in limited number of studies, using lipofection of cells and tumors with plasmids encoding reporter gene GFP and therapeutic gene iNOS under the control of p21 promoter [4,10-12]. Selective transcriptional targeting using the p21 promoter was demonstrated in an *in vitro* model of human microvascular endothelial cells (HMEC-1) and in an *ex vivo* rat tail arterial segment model [10]. Furthermore, tumor cell radio-sensitization *in vitro* and antitumor effectiveness *in vivo* were proven using different radiation regimes in murine fibrosarcoma (RIF-1) tumors and human colon adenocarcinoma (HT29) xenografts [11,12]. Another group demonstrated that p21 promoter driven therapeutic gene herpes simplex virus type-1 thymidine kinase (*HSVtk*) transduced by adeno-associated virus vector in the human breast cancer cells (MCF-7) can radio-sensitize the cells to repetitive treatment with low dose (1 Gy) irradiation [13].

To date p21 promoter was used in the context of the suicide gene therapy with therapeutic gens that have local effect [4,10-13], but lack systemic antitumor effect. One of the therapeutic genes that has already demonstrated its systemic radio-sensitizing effect is a gene that encodes for the secretory protein interleukin 12 (IL-12). IL-12 is a heterodimeric pro-inflammatory cytokine with multiple functions, including the induction of interferon-γ (IFN-γ), activation of T helper and NK cells [14,15], and anti-angiogenic activity [16-18]. Recombinant IL-12 was proven to have potent antitumor and antimetastatic effects against murine tumors [19], yet its clinical application was hindered by dose-limiting toxicity associated by its systemic administration [20,21]. Systemic treatment was therefore canceled to be replaced with IL-21 gene therapy, which has already reached the clinical phase [22-24]. To avoid systemic toxicity clinical trials have been designed to administer IL-12 directly to the tumor site, for instance by electroporation of IL-12 plasmid into the metastatic melanoma lesions [25]. The results of the phase I/II melanoma clinical trial demonstrated [24] the safety of this approach and also clinical response in the treated and distant non-treated metastases.

The main aim of our study was to evaluate anti-tumor effectiveness of combined radio-gene therapy with the plasmid encoding therapeutic gene *Il*-*12* under the control of the inducible p21 promoter in a mouse mammary adenocarcinoma tumor model. For this purpose we first tested the suitability of the p21 promoter for the radiation induced transcriptional targeting using different reporter gene experimental models by determining the induction of expression of reporter gene under the control of p21 promoter. Specific combination of the p21 inducible promoter and the radio-sensitizing therapeutic gene *Il-12* with radiotherapy has not been tested before. In addition, clinically used electrotransfer of plasmid DNA was employed in our study and the study was extended to another tumor model, mammary carcinoma, which has not been tested yet by transcriptional targeting using p21 promoter. We showed that p21 promoter is suitable for interleukin 12 radiation induced transcriptional targeting in a mouse mammary adenocarcinoma.

## Results

### p21 promoter is inducible with irradiation

**Figure 1:**
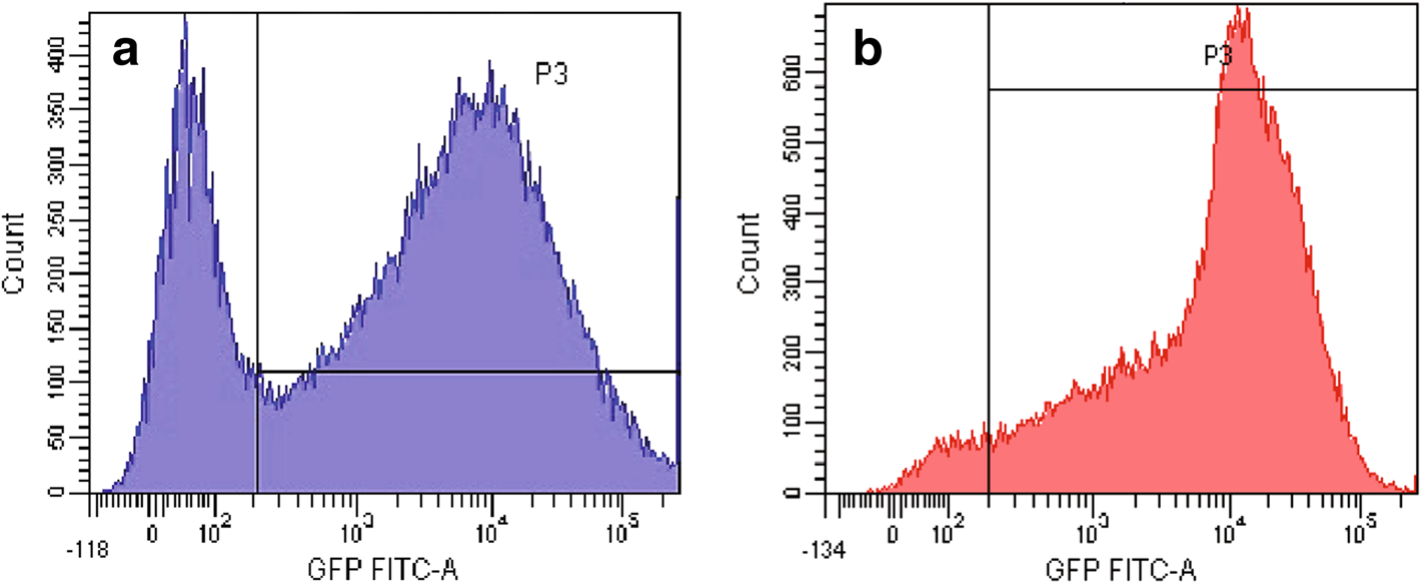
**Flow cytometry histograms of GFP expression in (a) TS/A CMV**-**EGFP and (b) TS/A p21-EGFP cell line.**

**Figure 2:**
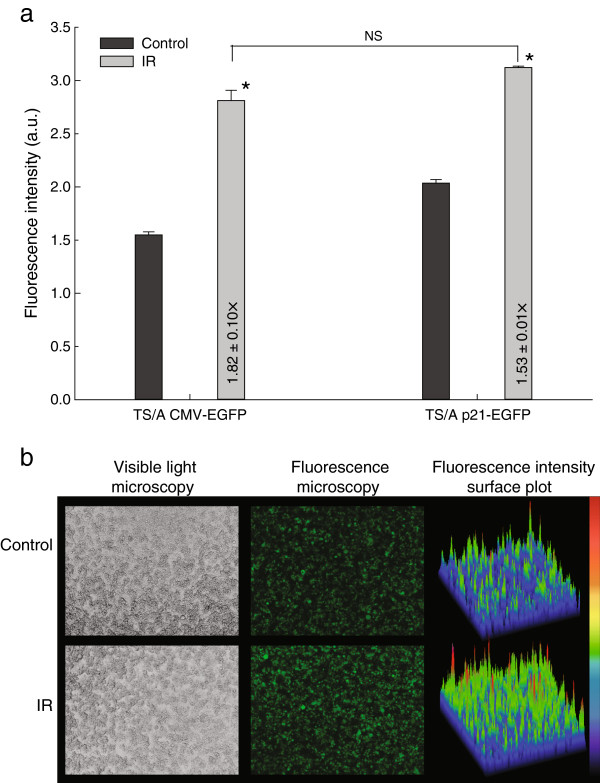
**Induction of reporter gene expression *in vitro*. (a)** Fluorescence intensity in TS/A cell lines stably transfected either with p21 (p21-EGFP) or constitutive promoter (CMV-EGFP) driven reporter gene GFP after 6 Gy irradiation (IR). Numbers in the bars are factors of induction of reporter gene expression or fold-induction, calculated by dividing the fluorescence obtained in the induced group by the fluorescence in the control group. The data were pooled from three independent experiments performed in 12 replicates and are presented as means + SEM; *, P < 0.05; NS, non-significant, **(b)** Visible and fluorescent images and surface plots of the TS/A cells stably transfected with p21 driven reporter gene GFP after 6 Gy irradiation (IR). On the surface plots fluorescence intensities are represented linearly on a rainbow scale with red being the maximum signal and black being the lowest signal.

The suitability of the p21 promoter for the radiation induced transcriptional targeting was tested using *in vitro* and *in vivo* reporter gene experimental models (stably transfected cell lines, stably transfected tumors and transiently transfected muscles). Fluorescence was determined by fluorescence micro-plate reader *in vitro* and by non-invasive fluorescence imaging *in vivo* and factors of induction of reporter gene expression after irradiation were calculated by dividing the fluorescence obtained in the induced group by the fluorescence in the control group. Stably transfected cell lines expressing the GFP reporter gene under the control of the p21 or a constitutive CMV promoter were successfully prepared (see methods) and designated as TS/A p21-EGFP and TS/A CMV-EGFP, respectively. Higher percentage of GFP expressing cells (95%) was demonstrated by flow cytometry in TS/A p21-EGFP cell line than in TS/A CMV-EGFP cell line (70%) (Figure 1). Irradiation of cells with 6 Gy significantly upregulated GFP expression in both cell lines (p < 0.05) (Figure 2).

**Figure 3:**
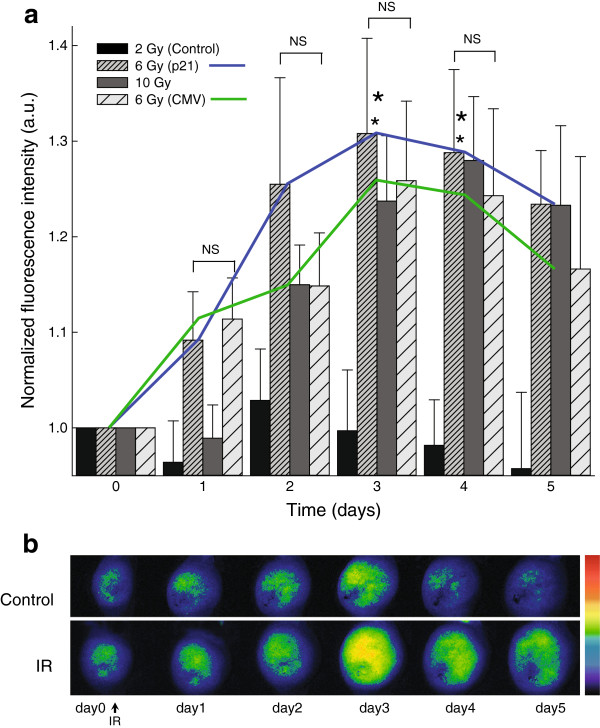
**Induction of reporter gene expression *in vivo *(tumors). (a)** Bar chart: normalized fluorescence intensity in the stably transfected TS/A p21-EGFP tumor model with reporter gene GFP under the control of p21 promoter after 0, 2, 6 and 10 Gy irradiation, and in TS/A CMV-EGFP tumor model with reporter gene under the control of CMV promoter after 6 Gy irradiation. For all experimental groups fluorescence is expressed in arbitrary units and is normalized on the day 0 and on the appropriate controls: for the p21 promoter on the non-irradiated TS/A p21-EGFP tumors and for the CMV on the non-irradiated TS/A CMV-EGFP tumors. The data were pooled from 2 independent experiments with 4–5 animals in each experimental group and are presented as means + SEM; *, P < 0.05 vs. control; NS, non-significant. Line plot: comparison of the induction dynamics of the inducible p21 and constitutive CMV promoter after 6 Gy irradiation. **(b)** Images of stably transfected TS/A p21-EGFP tumors in control group and experimental group that received 6 Gy irradiation (IR). Fluorescence intensities are represented linearly on a rainbow scale with red being the maximum signal and black being the lowest signal.

Stably transfected tumors were induced in BALB/c mice by injection of stably transfected TS/A CMV-EGFP and TS/A p21-EGFP cells. TS/A p21-EGFP tumor model was used to test dose response of the promoter using tumor irradiation with 2, 6 and 10 Gy. The results demonstrated that the induction was not dose dependent, since the dose of 6 Gy induced higher reporter gene expression than dose of 10 Gy. Increase in fluorescence intensity was the highest and statistically significant compared to the control group (p < 0.05), on the third day after 6 Gy irradiation by a factor of 1.16× (Figure 3). The activity of p21 promoter was compared to the activity of the constitutive CMV promoter in the stably transfected TS/A CMV-EGFP tumor model. The activity of both promoters after 6 Gy irradiation was very similar; the induction of p21 promoter was slightly more rapid and higher, but the differences were not statistically significant (p > 0.05) (Figure 3).

**Figure 4:**
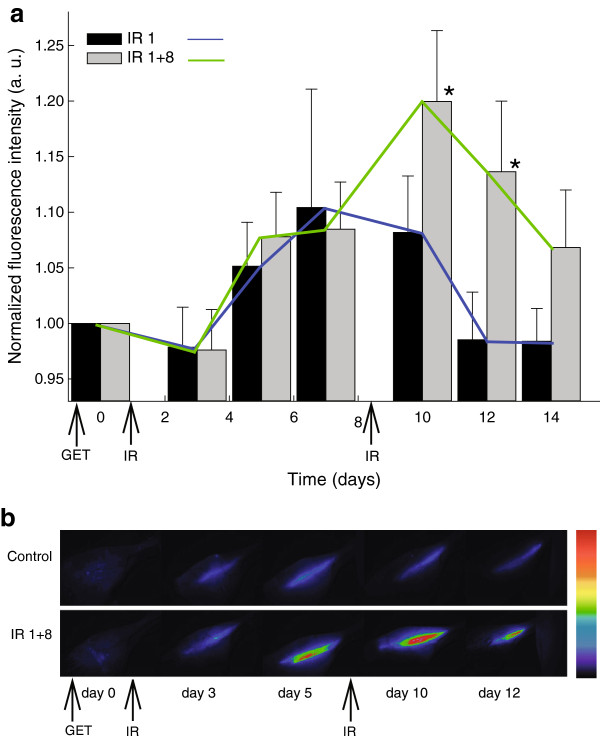
**Induction of reporter gene expression *in vivo *(muscles). (a)** Bar chart: normalized fluorescence in the mouse muscles transiently transfected with the plasmid carrying reporter gene GFP under the control of p21 promoter after 6 Gy irradiation on day 1 and day 8 after gene electrotransfer (GET) of p21-EGFP plasmid. For all experimental groups fluorescence is normalized on the day 0 and is expressed in arbitrary units. The data were pooled from 2 independent experiments with 7–8 animals in each experimental group and are presented as means + SEM; *, P < 0.05 vs. control. Line plot: induction dynamics of the p21 promoter after 6 Gy irradiation on day 1 and day 8 after gene electrotransfer (GET). **(b)** Images of mouse legs in control group and experimental group that received 6 Gy irradiation on day 1 and day 8 after the transfection with plasmid p21-EGFP (IR 1 + 8). Fluorescence intensities are represented linearly on a rainbow scale with red being the maximum signal and black being the lowest signal.

Model of transiently transfected muscles was used to test if promoter can be activated after a transient transfection, and if promoter can be re-induced. Muscle irradiation with 6 Gy one day after the gene electrotransfer with plasmid p21-EGFP activated the reporter gene, but the induction was not statistically significant (p > 0.05), though significant increase in reporter gene expression (1.2×, p < 0.05) was obtained with the repeated muscle irradiation, 7 days after the first, providing evidence of reporter gene re-induction (Figure 4).

### Radio-gene therapy with inducible *Il*-*12* plasmid has a synergistic therapeutic effect

To determine the therapeutic effect of combined radio-gene therapy, TS/A tumors were transfected with plasmids encoding p21 or constitutive promoter-driven *Il*-*12*, using gene electrotransfer (GET), and 24 hours later irradiated with 6 Gy. Tumor growth was followed, and tumors and blood were collected 5 days after the irradiation to determine intratumoral and serum levels of IL-12 protein and intratumoral concentrations of *Il*-*12* mRNA.

**Figure 5:**
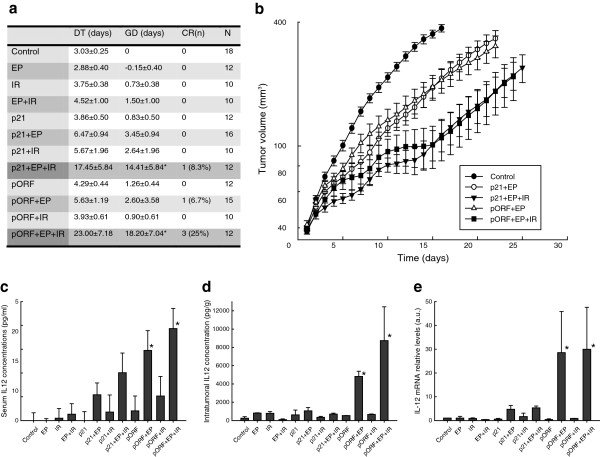
**Therapeutic effect of radioinducible IL-12 gene therapy after 6 Gy irradiation in mouse TS/A tumors.** EP, electroporation; IR, irradiation; p21, intratumoral injection of the p21-mIL-12 plasmid; p21 + EP, gene electrotransfer of the p21-mIL-12 plasmid; p21 + EP + IR, radio-gene therapy with the p21-mIL-12 plasmid; pORF, intratumoral injection of the pORF-mIL-12 plasmid, pORF + EP, gene electrotransfer of the pORF-mIL-12 plasmid; etc. **(a)** DT, doubling time i.e. time needed for the tumor to double in size; GD, growth delay i.e. difference between the doubling time of the specific experimental group and average doubling time of the control groups); CR, complete response i.e. complete disappearance of the tumor lasting for 100 days; N, number of the animals in the individual experimental group; data are presented as mean ± SEM;*, P < 0.05 vs. control. **(b)** Tumor growth curves after radioinducible IL-12 gene therapy. The data were pooled from 2 independent experiments with 18–10 animals in each experimental group and are presented as means with the standard errors of the mean. **(c)** Serum and **(d)** intratumoral IL-12 protein concentrations and **(e)** intratumoral *Il-12* mRNA levels on the 5^th^ day after the induction with irradiation. Each column represents mean + SEM. **(c)** *, P < 0.05 vs. control, 6–9 animals per experimental group. **(d)** *, P < 0.05 vs. all experimental groups except pORF + EP, 2–3 animals per experimental group. **(e)** *, P < 0.05 vs. control, 2–3 animals per experimental group.

Antitumor effect of radio-gene therapy with *Il*-*12* under control of the inducible p21 promoter was comparable to effect of the same therapy using a constitutive promoter. Tumor growth delay was statistically significantly longer, compared to control group, in both experimental groups that received radio-gene therapy with inducible and constitutive IL-12 plasmids (p < 0.05) (Figure 5). Although the growth delay was longer in the group with the constitutive promoter (18.2 days) than in the group with inducible promoter (14.4 days) the difference was not statistically significant (p > 0.05). All other pertinent control groups did not result in significantly prolonged growth delays, compared to the untreated control group. The growth delays in both therapeutic groups (receiving radio-gene therapy with inducible or constitutive IL-12 plasmids) were longer than the sum of radio- (0.7) and gene-monotherapies (3.45), indicating that the effect of combined therapy was synergistic. The synergistic effect was also confirmed by criteria for assessment of combined effect of two therapies with independent mechanisms of action [26]. Furthermore, tumor cures were obtained after radio-gene therapy using both plasmids.

Intratumoral and serum concentrations of IL-12 protein, as well as *Il*-*12* mRNA, were statistically significantly elevated in the group that received radio-gene therapy with the constitutive pORF-mIL-12 plasmid (Figure 5). Furthermore, serum concentrations were statistically significantly elevated in groups that received GET of the constitutive pORF-mIL-12 plasmid without irradiation. Elevated serum concentrations were also apparent, but not statistically significant, after GET of inducible plasmid p21-mIL-12, especially in combination with irradiation.

## Discussion

To improve the efficacy and safety of gene therapy spatial and temporal regulation of therapeutic gene is needed. This kind of regulation is especially important for the clinical application of gene therapy to achieve a sustained therapeutic level of transgene product without systemic toxicity and also to be able to adjust the transgene expression according to the stage of disease. Although gene therapy is becoming a realistic alternative modality for treatment of cancer with clinical trials underway [27], it is not very likely that it will be used as a monotherapy. If gene therapy is combined with radiotherapy, a well-established cancer treatment, inducible promoters can be used to gain more controlled transgene expression in an approach called radiation induced transcriptional targeting.

In the first part of our study, the feasibility of the p21 promoter for inducible therapy was tested using *in vitro* and *in vivo* reporter gene experimental models with GFP. Selection of irradiation doses (2, 6 and 10 Gy) to test the dose response of the p21 promoter *in vivo* was based on established radiobiology protocols [28]. Reporter gene experimental models were prepared by stable transfection of the tumor cells to ensure higher and more uniform gene expression (standardized conditions) enabling more reproducible data and lower intra- and inter- variability among experimental groups. This experimental setup was also established to comply with 3Rs’ rule in animal experimentation (EU directive 2010/63/EU). Furthermore, established experimental models enabled us to determine the activity of promoters by simply quantifying the fluorescence intensity, presuming that fluorescence is proportional to gene expression that is proportional to the activity of the upstream promoter. Interestingly, in a similar study also using p21 promoter driven GFP reporter gene [10], expression was determined with a semi quantitative Western blot method, instead of fluorescence intensity. The induction factors after radiation were relatively low compared to factors obtained in that study [10]. However, similar to our results, the induction factor was the highest after the irradiation with 6 Gy [10], which is in agreement with our dose response experiment on stably transfected tumors. The reason for low induction factors in our study could be that in our study a cancer cell line (TS/A) was used and cancer cell lines in general have high basal level activity of p21 promoter, compared to non-cancer cell lines, with characteristically low basal level activity [4]. Namely, the induction factors appear to be different for different cell lines; the differences are especially apparent between normal and cancer cell lines [4]. Already during the preparation of the stable cell line with p21 promoter we noticed that basal activity of the p21 promoter was relatively high as it was easily seen under the fluorescence microscope, and was comparable to the intensity in cell lines with CMV promoter. Generally, high basal activity is an unwanted characteristic in an inducible promoter. Nevertheless, if p21 promoter is to be used in cancer therapy, this selectiveness is an advantage and not hindrance because it enables selective targeting of cancer cells, which could be highly beneficial.

Another, already proven and potentially beneficial, characteristic of p21 promoter is that it can be induced by hypoxia [12] which is a physiological feature of almost all solid tumors [29]. Hypoxia remains a great hindrance for radiotherapy, since oxygen is essential for generation of ROS that are responsible for therapeutic efficacy of irradiation [30,31]. The physiological difference in the oxygenation can, on the other hand, be exploited for more selective cancer therapy with the use of hypoxia inducible promoters [32]. Since hypoxia inducible gene therapy relies on the lack of oxygen and radioinducible therapy needs the production of oxygen derived free radical, neither approach is adequate for the treatment of the whole tumor. Vectors containing synthetic promoters responsible to both stimuli have therefore been developed [33]. These so called chimeric promoters that contain an optimal number and arrangement of responsive elements derived from different hypoxia and radioinducible promoters were initially introduced in 2002 [34]. In the more advanced of the chimeric promoters the control over the expression of transgene moves from the inducible to a strong constitutive promoter after the initial signal in order to ensure higher level of expression [35]. In addition to this so called “molecular amplification switch” vectors with these promoters also have to contain a so called “stop cassette”, that halts the expression of the transgene in the absence of a stimuli [36]. Although p21 promoter is a simple native promoter of a gene that is present in all human cells, it can be matched-up with these sophisticated synthetic promoters because it has similar characteristics: it is induced by hypoxia and irradiation, it has a high basal activity that is limited to tumor cells, and after the removal of a stimuli its activity in normal cell falls again to the basal level, but remains high in tumor cell.

In our study the activity of inducible p21 promoter was compared to the activity of the CMV promoter which is a standard promoter used in many gene therapy studies [37], although we were fully aware of its drawbacks. Namely, we demonstrated that CMV promoter was also induced after irradiation: *in vitro* the induction was even higher in the cell line with CMV promoter than in cell line with p21 promoter, although the difference was not statistically significant. *In vivo* we also showed that the induction dynamics were similar in both inducible p21 and constitutive CMV promoter; the induction of p21 promoter was slightly faster and more pronounced but the difference was not statistically significant. We tackled the problem of the non-constitutive nature of CMV promoter in a separate study, where we showed that CMV promoter indeed gets activated after irradiation, which limits its usefulness as a constitutive promoter [38].

At the end of reporter genes part of the study we wanted to check if p21 promoter is suitable for inducible therapy also after transient transfection, since this kind of therapy is more appropriate for clinical use of gene therapy. For the model, transiently transfected muscles model was chosen, because it allows better detection of fluorescent reporter gene detection then the tumor model. In addition to higher transfection efficiency, longer lasting transfection is characteristic for skeletal muscles, therefore reinducibility of p21 promoter could be determined. This could not be done on stably transfected tumors or tumor cells because of their short life span due to the fast growth rate of cells and tumors. Skeletal muscle was proven in the past as a good tissue for electrically assisted gene transfer [39-41]. We demonstrated that p21 promoter can be induced and re-induced in the transiently transfected muscles; deducted from the level of expression after the first and the second induction alone, the combined induction was the sum of both. To our best knowledge this is the first time p21 promoter was used in a similar experiment and proved that it is suitable for transcriptional targeting after transient transfer in the muscle tissue.

In the second part of the study the therapeutic effectiveness of the combined radio-gene therapy with p21 promoter controlling the expression of the therapeutic gene *Il-12* was evaluated in a relatively radioresistant and IL-12 resistant TS/A tumor model [42,43]. The radiation induced transcriptional targeting approach allows for any therapeutic gene with radiosensitizing properties to be chosen. One of the therapeutic genes that has already demonstrated its radio-sensitizing effect is IL-12 [44-52]. Although not fully elucidated, proposed mechanisms of IL-12 radio-sensitization were enhanced tumor antigen presentation due to radiation induced apoptosis [49], anti-angiogenic effects [42], and the production of radiosensitizer nitric oxide [52]. In addition to potent local radio-sensitizing activity [44-52], IL-12 gene therapy can also offer the systemic protection against distant metastases by induction of an effective immune response against tumor antigens or inhibiting angiogenesis of metastases [21,24,25,53].

The results of this part of our study showed that radio-gene therapy with *Il*-*12* under control of the inducible p21 promoter had a good antitumor effect that was comparable to that of the same therapy using a constitutive promoter. Furthermore, the effect of combined therapy proved to be synergistic to both radio-and gene- therapy. Synergistic working of IL-12 and irradiation was reported before in a number of preclinical studies combining local or systemic (through systemic IL-12 release from the transfected muscle) IL-12 gene therapy with local irradiation in several different murine tumors [44-48]. In one such study, that is perhaps the most similar to ours, a plasmid in which the expression of IL-12 was controlled by the inducible Egr1 promoter was used in the B16 murine tumor model [54]. The subcutaneous tumors were injected with naked plasmid DNA and 24 later exposed to 2 and 5 Gy irradiation and this treatment protocol was then repeated 3-times per day every second day. The result was a statistically relevant tumor growth delay and elevated intratumoral IL-12 level in the group that received this triple radiogene therapy compared to the control group.

Although therapeutic effect of radio-gene therapy with the inducible p21 promoter was comparable to that of the same therapy using the constitutive promoter, serum and intratumoral concentrations of IL-12 and intratumoral *Il-12* mRNA levels were significantly lower after radio-gene therapy with p21 promoter then after radio-gene therapy with the constitutive promoter. Lack of correlation between serum and tumoral levels of IL-12 and antitumor effect was also observed in previous studies. Specifically, the levels of IL-12 vary significantly among different experiments and different mouse models indicating that the therapeutic effect of the IL-12 gene therapy cannot be directly linked to the IL-12 protein or mRNA levels [47,55,56]. Moreover, elevated levels may not even be prerequisite for the therapeutic effect [56]. Another possible reason for the observed difference in our study could be that we determined the levels at day 5 after radio gene [47]. Since the therapeutic effect of both constitutive and p21 plasmids were similar, we assume that we probably missed the peak of *Il-12* expression in p21 group. This is also supported by the results of the reporter gene part of our study, where we showed that the expression of GFP controlled by p21 promoter was the highest on the third day after induction with irradiation. Considering that GFP’s half-time [57] is noticeably longer than IL-12-s half-time [58], we speculate that peak in *Il-12* expression after radio gene therapy with p21 promoter occurred before the measurements.

Based on the results of this preclinical study showing that p21 promoter has a similar effectiveness as the constitutive promoter, we trust it is suitable for further development. Namely, general tendency in gene therapy is to replace virus derived promoters with endogenous promoters, due to the safety concerns and longer lasting expression they support [37]. Therefore, p21, as an endogenous promoter with low basal activity in normal cells, higher activity in cancer cells and inducibility by different treatment induced stresses, is a prospective candidate for translational studies in cancer gene therapy.

## Conclusions

In this study we demonstrated, using the reporter gene experimental models, that p21 promoter is a feasible candidate for radiation induced transcriptional targeting. As a proof of principle, the combined radio-gene therapy with inducible *Il*-*12* plasmid was demonstrated to have a synergistic therapeutic effect to radio and gene-monotherapies, making this approach feasible for the combined treatment with radiotherapy, which, we believe, is crucial for translation of this approach into the clinical setting.

## Methods

### Plasmids

Two commercial available plasmids were used: a plasmid encoding green fluorescence protein (GFP) under the control of the strong constitutive human cytomegalovirus (CMV) immediate early promoter and neomycin resistance gene (pEGFP-N1, Clontech, Basingstoke, UK) and a plasmid encoding mouse *Il*-*12* under the control of a constitutive hybrid promoter EF-1α/HTLV (pORF-mIL-12, Invivogen, Tolulouse, France). Source plasmid for p21 promoter sequence was a plasmid encoding *GFP* under the control of the human p21 promoter (and neomycin resistance gene) (p21-EGFP) which was a kind gift from Irena Hreljac, (National Institute of Biology, Ljubljana, Slovenia). Eukaryotic expression plasmid encoding the therapeutic gene *Il*-*12* under the control of p21 promoter (p21-mIL-12) was prepared from plasmids p21-EGFP and pORF-mIL-12 using standard molecular-biological methods of restriction and ligation: first the reporter gene GFP was cut out of the plasmid p21-EGFP by the restriction enzymes *Sal*I and *Xba*I and was then replaced with the therapeutic gene *Il*-*12*, which was cut out of the plasmid pORF-mIL-12 using the compatible restriction enzymes *Sal*I and *Nhe*I. For the experiments plasmid DNAs were isolated using the EndoFree Plasmid Mega Kit (Qiagene, Hilden, Germany) according to manufacturer’s instructions and diluted in endotoxin free water to a concentration of 1 mg/ml. Plasmid DNA concentration and pureness was determined spectrophotometrically and by gel electrophoresis.

### Cell lines and experimental animals

Murine adenocarcinoma of the mammary glands TS/A [41] cell line was used in the experiments. During the *in vitro* experiments, cells were maintained in Eagle’s minimum essential medium (EMEM, Sigma, Taufkirchen, Germany), supplemented with 10% fetal calf serum (FCS, Sigma, Taufkirchen, Germany), 2 mM L-glutamine (Gibco Invitrogen, San Diego, California, USA) and 100 IU/ml penicillin/streptomycin (Pliva, Zagreb, Croatia) in a humidified incubator at 37°C with 5% CO_2_.

For the *in vivo* experiments, female BALB/c and C57BL/6 mice obtained from the Institute of Pathology, Faculty of Medicine, University of Ljubljana, Slovenia, were used. At the beginning of the experiments, the animals were 10–12 weeks old. Mice were housed and maintained in a specific pathogen-free animal colony at constant room temperature (21°C) and 12 h light/dark cycle. Food and water was provided ad libitum. Animals were subjected to an adaptation period of 7–10 days before the experiments were carried out. All procedures on animals were performed in accordance with the official guidelines of the EU directive (2010/63/EU) and with the permission of the Ministry of Agriculture and environment of the Republic of Slovenia (permission No.: 323-02-632/2005/6).

### Reporter gene experimental models

#### Stable cell lines

TS/A cells were transfected with the p21-EGFP (containing the p21 promoter) and pEGFP-N1 (containing the CMV promoter) plasmids. Electroporation was used for introduction of plasmid DNA into cells. Specifically, cells grown as a monolayer were harvested and a 2.5 × 10^7^ cells/ml cell suspension was prepared in electroporation buffer (125 mM sucrose, 10 mM K_2_HPO_4_, 2.5 mM KH_2_PO_4_, 2 mM MgCl_2_ × 6 H_2_O). A dense cell suspension with a concentration of 1 × 10^6^ cells and 10 μg of pEGFP-N1 in 50 μl of electroporation buffer was placed between two flat parallel stainless steel electrodes with a 2 mm gap connected to the GT-1 electroporator (University of Ljubljana, Faculty of Electrical Engineering, Ljubljana, Slovenia) and subjected to eight square-wave electric pulses with an amplitude per distance ratio 700 V/cm, 5 ms duration time and 1 Hz repetition frequency. After electroporation cells were incubated for 5 min at room temperature, plated into Petri dishes and then cultured for two months under increasing concentrations (1200–2000 μg/ml) of the selection agent geneticin (Gibco Invitrogen, San Diego, California, USA) to obtain resistant clones. Clones with the highest GFP expression were identified by fluorescence microscopy (Olympus, Hamburg, Germany), isolated, propagated and frozen in liquid nitrogen for subsequent experiments. To determine the number of fluorescent cells, flow cytometry analysis of stable cell lines carrying p21 and CMV promoter-driven reporter gene constructs was performed: cells were trypsinized, collected and 2 × 10^4^ cells from each stable cell line were analyzed using flow cytometry (Becton Dickinson, Calibur, Franklin Lakes, USA) by determining the percentage of EGFP-positive (fluorescent) cells and median fluorescence intensity of the EGFP (Figure 1). Laser excitation was 488 nm and number of events was 20.000.

#### Stably transfected tumors

2 × 10^6^ viable TS/A CMV-EGFP and TS/A p21-EGFP tumor cells prepared from cell cultures in vitro were injected dorsolaterally in BALB/c mice for the induction of solid subcutaneous tumors. When the tumors reached approximately 40 mm^3^ in volume (7–10 days), mice were randomly divided into experimental groups and subjected to a specific experimental protocol.

#### Transiently transfected muscles

C57Bl/6 mice were anaesthetized with isofluran (Torrex Chiesi GmbH, Wien, Austria) using an isoflurane vaporizer (Datex Ohmeda, Helsinki, Finland). Plasmid pEGFP-N1 (20 μg in 20 μl of water) was injected into both right and left musculus tibialis cranialis with a thin (26 G) needle. The hind legs were placed between two flat parallel stainless steel electrodes with rounded corners (dimensions 20 mm × 10 mm) with a 6 mm gap between the electrodes connected to the electric pulse generator Cliniporator™ (IGEA s.r.l., Carpi, Italy), and subjected to one high-voltage square-wave electric pulse with an amplitude per distance 600 V/cm and 100 μs duration and 4 low-voltage square-wave electric pulses with an amplitude per distance 80 V/cm, 100 ms duration and 1 Hz repetition frequency. Good contact between the electrodes and legs was assured by hair removal using hair removal cream (Vitaskin, Krka, d.d., Novo mesto, Slovenia) and use of a conductive gel (Kameleon d.o.o., Maribor, Slovenia). A day after gene electrotransfer, mice were randomly divided into experimental groups and subjected to a specific experimental protocol.

### Irradiation

Cells, tumor-bearing BALB/c mice and C57BL/6 mice with transiently transfected muscles were irradiated using an X-ray unit Darpac 2000 (Gulmay Medical Ltd, Shepperton, UK) operating at 220 kV, 10 mA, and with 0.55 mm Cu and 1.8 mm Al filtration. Stably transfected cells were plated at a density of 1.7× 10^4^ cells/cm^2^ and were irradiated with single dose of 6 Gy. Mice with stably transfected tumors were irradiated with 2, 6 and 10 Gy and mice with transiently transfected muscles were irradiated with the dose of 6 Gy at day 1 and day 8 after the gene electrotransfer. During irradiation, mice were restrained in special lead holders with apertures for irradiation of the tumors/legs, exposing only the tumors/legs and shielding the rest of the body from irradiation.

### Quantification of reporter gene expression

***In vitro:*** Three days after the treatments, cells were trypsinized, collected and 2 × 105 cells were plated in 96-well microplates. Expression of the reporter gene was followed using the fluorescence microscopy (Olympus, Hamburg, Germany) and determined using the fluorescence microplate reader Infinite 200 (Tecan, Männedorf, Switzerland). Induction factors i.e. factors of induction of reporter gene expression after irradiation were calculated as quotients between induced and control group.

***In vivo*** non-invasive fluorescence imaging: After the treatments, fluorescence intensity of the tumors and muscles expressing GFP was followed transcutaneously using a fluorescence stereo microscope (Lemar.V.12., Zeiss, Jena, Germany), which enabled non-invasive follow-up of the intensity and duration of GFP expression. At each observation under the microscope, hair over the tumor or muscle was removed using an electric shaver and hair removal cream and animals were anaesthetized with isoflurane as described above. Digital images of fluorescence were recorded everyday post-treatment for 8 days for tumors and every 2–3 days for 12 days in the case of the muscle with a digital color camera (Axiocam MRc5, Zeiss, Jena, Germany) connected to the fluorescence stereo microscope. During capture, tumors or legs were placed in a special holder to minimize the movement of animals caused by breathing and to ensure the same positioning at each observation.

Fluorescent image of the tumor with stably transfected tumor cells and muscle fibers fluorescing through the skin were analyzed using the ImageJ software tool (National Institute of Mental Health, Research Services Branch, Bethesda, Maryland, USA). Images of the same tumor or muscle taken at different time points were stacked together. Fluorescence of the tumor cell or muscle fibers was separated from the background fluorescence using the threshold tool and mean grey value i.e. intensity of the area under threshold was determined. Adjusted mean fluorescence intensity of each tumor or muscle slice in the stack was then normalized to the mean fluorescence intensity at day 0.

### Radio-gene therapy

Radio-gene therapy was performed in two steps consisting of gene electrotransfer to tumors followed by irradiation. For the execution of gene electrotransfer, the tumors were first injected with 50 μg of plasmid DNA (p21-mIL-12 or pORF-mIL-12) in 50 μl of sterile water and 10 minutes later subjected to 8 square-wave electric pulse with amplitude per distance 600 V/cm and 5 ms duration with 1 Hz repetition frequency. Good contact between the electrodes and tumor was assured by hair removal using electric shaver and use of a conductive gel (Kameleon d.o.o., Maribor, Slovenia). Twenty-four hours later tumors were irradiated with a dose of 6 Gy as described above.

### Determination of therapeutic effect

The therapeutic effect of radio-gene therapy was determined using the tumor growth delay assay. Tumors were measured in three perpendicular directions (a, b, c) every 2–3 days using a digital caliper. Tumor volume was calculated by the formula: V = a × b × c × π/6. Tumor growth was followed until tumors reached average volume of 350 mm^3^, and then the animals were sacrificed. Animals with tumors in regression were examined weekly for the presence of the tumor for 15 consecutive weeks. The animals were considered cured if they were tumor-free at day 100. Doubling time (DT) or tripling time (TT) for each tumor was determined as the time when tumors reached double or triple the volume on day 0, respectively, and was expressed in days. Growth delay (GD) for each experimental group was determined as the difference between DT or TT of the experimental group and DT or TT of the control group.

### Tumors and blood collection for Enzyme-Linked ImmunoSorbent Assay (ELISA) and Real Time Polymerase Chain Reaction (qPCR)

Tumors and blood samples were collected from individual mice from each experimental group at day 5 post-treatment. Blood was collected from the intraorbital sinus into collection tubes and stored at room temperature for 2 hours. Serum was extracted from blood samples by centrifugation at 3,000 rpm for 5 min and immediately stored at −80°C for the ELISA test. Immediately after the blood was collected mice were sacrificed and tumors were removed, weighed and stored at −80°C. Frozen tumors were mechanically macerated in liquid nitrogen. For the ELISA test, tumor samples were diluted with 500 μl of PBS containing protease inhibitors (Protease Inhibitor Cocktail, phenylmethylsulfonyl fluoride (PMSF) and Sodium Orthovanadate, all Santa Cruz Biotechnology, Inc., Heidelberg, Germany, 10 μl of each per ml of PBS), thoroughly mixed and centrifuged for 10 min at 10,000 rpm. The supernatant was separated from the sediment and stored at −80°C until analysis. Both sets of samples (serums and supernatants from the tumor tissue) were analyzed using ELISA Quantikine Mouse IL-12 p70 Immunoassay (R&D Systems, Minneapolis, MN, USA) for detection of IL-12. Concentrations of IL-12 were calculated as pg of cytokine per ml of serum or ng of cytokine per mg of tumor tissue.

For the qPCR total RNA was extracted from frozen tumors using TRIzol Plus RNA Purification Kit (Invitrogen) according to the manufacturer’s instructions. The RNA concentrations were quantified by spectrophotometer at 260 nm. A 1 μg of total RNA was reverse transcribed into complementary DNA using SuperScript VILO cDNA Synthesis Kit (Invitrogen), according to manufacturer’s instructions. After reverse transcription, 2 μl of the 10-times and 100-times diluted mixture was used as the template for the following QPCR using TaqMan Gene Expression Master mix (Applied Biosystems) and TaqMan Gene Expression Assay (Applied Biosystems). TaqMan Gene Expression Assay contained pair of primers and TaqMan MGB probe (ID: Mm00434165_m1) to amplify the alpha subunit of IL-12 and TaqMan MGB probe (ID: Mm00446968_m1) to amplify the housekeeping gene *hypoxanthine*-*guanine phosphoribosyltransferase* (*Hprt1*), which was used as a reference gene. A total of 50 cycles of PCR was performed as follows: activation of AmpliTaq Gold Enzyme (10 min at 95°C), denaturation (15 s at 95°C), annealing and extension (1 min 60°C). The PCR products were analysed using 7300 System SDS software V.1.3.1 (Applied Biosystems). The level of *Il*-*12* expression in each sample was calculated as ratio of *Il*-*12* vs. reference gene *Hprt1* RNA and normalized to untreated control group.

### Statistical analysis

Statistical analysis was performed using SigmaPlot 12.0 (Systat Software Inc., San Jose, CA, USA). The data were tested for normality of distribution using the Shapiro-Wilk test. Differences between independent experimental groups were statistically evaluated by the Student’s *t* test and differences between dependent experimental groups by one-way analysis of variance (one-way ANOVA). A P value of less than 0.05 was considered to be statistically significant. The synergistic effect between treatments was determined by criteria for assessment of combined effect of two therapies with independent mechanisms of action [26].

## Competing interests

The authors declare that they have no competing interests.

## Authors’ contributions

All authors have made substantial contribution to the study presented in the manuscript. UK contributed to the design of the study, performed most of the acquisition, analysis and interpretation of the data, and wrote the manuscript. GS and MC made substantial contribution to the conception and design of the study, interpretation of the data, and revise the manuscript draft critically. All authors read and approved the final manuscript.
